# Scrofula: an uncommon tuberculosis manifestation reappearing in Italy

**DOI:** 10.1093/jscr/rjaf1035

**Published:** 2026-02-27

**Authors:** Gerardo D’Amato, Mario Musella, Carolina Bartolini, Chiara Bellantone, Lucrezia Borrelli, Alessandra D’Ambrosio, Antonio Franzese, Vincenzo Schiavone, Mafalda Ingenito

**Affiliations:** Dipartimento di Scienze Biomediche Avanzate, Università degli Studi di Napoli “Federico II”, Via Sergio Pansini 5, Napoli, Campania 80131, Italy; Dipartimento di Scienze Biomediche Avanzate, Università degli Studi di Napoli “Federico II”, Via Sergio Pansini 5, Napoli, Campania 80131, Italy; Dipartimento di Scienze Biomediche Avanzate, Università degli Studi di Napoli “Federico II”, Via Sergio Pansini 5, Napoli, Campania 80131, Italy; Dipartimento di Nutrizione Clinica e Diabetologia, Ospedale San Carlo di Nancy—GVM, Via Aurelia 275, Roma, Lazio 00165, Italy; Dipartimento di Scienze Biomediche Avanzate, Università degli Studi di Napoli “Federico II”, Via Sergio Pansini 5, Napoli, Campania 80131, Italy; Dipartimento di Scienze Biomediche Avanzate, Università degli Studi di Napoli “Federico II”, Via Sergio Pansini 5, Napoli, Campania 80131, Italy; Dipartimento di Scienze Biomediche Avanzate, Università degli Studi di Napoli “Federico II”, Via Sergio Pansini 5, Napoli, Campania 80131, Italy; Dipartimento di Scienze Biomediche Avanzate, Università degli Studi di Napoli “Federico II”, Via Sergio Pansini 5, Napoli, Campania 80131, Italy; Dipartimento di Scienze Biomediche Avanzate, Università degli Studi di Napoli “Federico II”, Via Sergio Pansini 5, Napoli, Campania 80131, Italy

**Keywords:** scrofula, cervical tuberculosis lymphadenitis, tuberculosis, extrapulmonary tuberculosis

## Abstract

Cervical tuberculous lymphadenitis (scrofula) is the most frequent form of extrapulmonary tuberculosis and may mimic malignant or other infectious conditions, especially in elderly patients or those with a prior oncological history. We report the case of an 88-year-old male with a history of treated lymphoma and chronic anticoagulation for atrial fibrillation, who presented with a rapidly enlarging cervical mass initially suggestive of malignancy. Cervical ultrasound revealed lymphadenopathies with no thyroid involvement. Fine-needle aspiration cytology (FNAC) excluded carcinoma and demonstrated caseous-purulent material. Microbiological and cytological analyses confirmed tuberculous lymphadenitis. Antitubercular therapy was initiated with progressive clinical improvement and no recurrence during follow-up. This case underscores the diagnostic challenges of scrofula, particularly in high-risk populations. FNAC is a valuable diagnostic tool, although repeat procedures or excisional biopsy may be required. Clinicians should maintain a high index of suspicion for tuberculosis in cases of atypical cervical lymphadenopathy to ensure timely diagnosis and treatment.

## Introduction

Tuberculosis (TB) is a chronic granulomatous infection caused by the Gram-positive, acid-fast bacilli of the genus *Mycobacterium*, caused especially by *Mycobacterium tuberculosis.* It remains a major public health problem worldwide for its ability to persist as a latent infection or manifest as a progressive disease [1]. While pulmonary TB is the most frequent form, the bacterium also has the potential to disseminate and affect nearly any organ system, including the bones, joints, lymph nodes, meninges, gastrointestinal tract, and genitourinary system. Cases of TB affecting sites other than the lungs are referred to as extrapulmonary TB (EPTB) [2].

Globally in 2023, ~ 8.2 million new TB cases were diagnosed and notified, of which 16% were extrapulmonary in nature [3]. Among the extrapulmonary manifestations, tuberculous lymphadenitis is the most common, with cervical tuberculous lymphadenitis (CTL), also known as scrofula, representing a frequent clinical presentation. CTL is regarded as a localized manifestation of systemic TB and may occur as a complication of primary infection, reactivation of dormant foci, or spread from a contiguous focus. It is often considered a diagnostic challenge, as its clinical and laboratory features can mimic other infectious and neoplastic conditions [4].

The diagnosis of CTL typically requires a combination of clinical history, physical examination, and laboratory investigations. Biopsy of the lymph node remains the gold standard, while fine-needle aspiration cytology (FNAC), acid-fast bacilli staining, and molecular tests such as polymerase chain reaction provide valuable adjunctive tools. A careful differential diagnosis is essential to distinguish tuberculous lymphadenitis from other causes of cervical lymphadenopathy, such as bacterial, viral, or malignant etiologies [5].

Given its close association with socioeconomic factors such as poverty, poor living conditions, and lack of awareness, the burden of CTL is especially relevant in TB-endemic regions, where late presentation and diagnostic delays are common.

In this report, we present a case of CTL, showing clinical presentation, diagnostic procedure, and management in accordance with our local guidelines. This case highlights the importance of early recognition and accurate differentiation of CTL to ensure timely treatment and favorable outcomes.

## Case report

**Figure 1:**
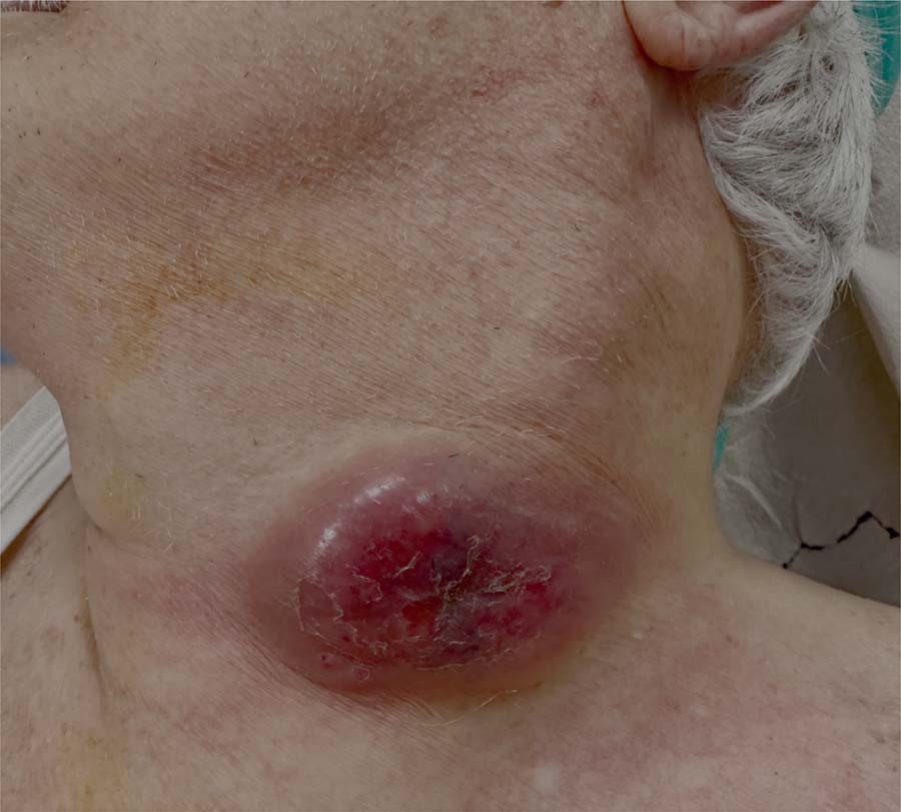
Preoperative clinical image of cervical tuberculous lymphadenitis (scrofula).

An 88-year-old male with a past medical history of treated lymphoma and chronic atrial fibrillation under long-term anticoagulation therapy presented with a rapidly enlarging cervical mass. The lesion had developed over a short period of time and was initially clinically suspected to represent a malignant process, particularly carcinoma, given the patient’s advanced age and oncological history (Fig. 1).

A neck ultrasound demonstrated multiple enlarged lymph nodes with internal liquefaction, consistent with necrotic transformation, but notably revealed no thyroid involvement or primary masses. Based on these findings, a FNAC was performed, which effectively excluded carcinoma, thereby reducing the likelihood of metastatic or primary thyroid malignancy.

Due to the persistence of diagnostic uncertainty and the atypical features of the lesion, a repeat fine-needle aspiration biopsy was undertaken. This procedure revealed caseous-purulent material, a hallmark finding strongly suggestive of scrofula. Subsequent cytological and microbiological analyses confirmed the diagnosis of CTL.

Following the definitive diagnosis, the patient was initiated on standard antitubercular therapy (ATT) in accordance with national guidelines [6]. The regimen consisted of an intensive phase with isoniazid, rifampicin, pyrazinamide, and ethambutol for 2 months, followed by a continuation phase with isoniazid and rifampicin for 4 months. Given the patient’s advanced age, history of lymphoma, and anticoagulant therapy, treatment was closely monitored with regular clinical, biochemical, and hematological assessments to minimize potential drug-related toxicities and interactions.

**Figure 2:**
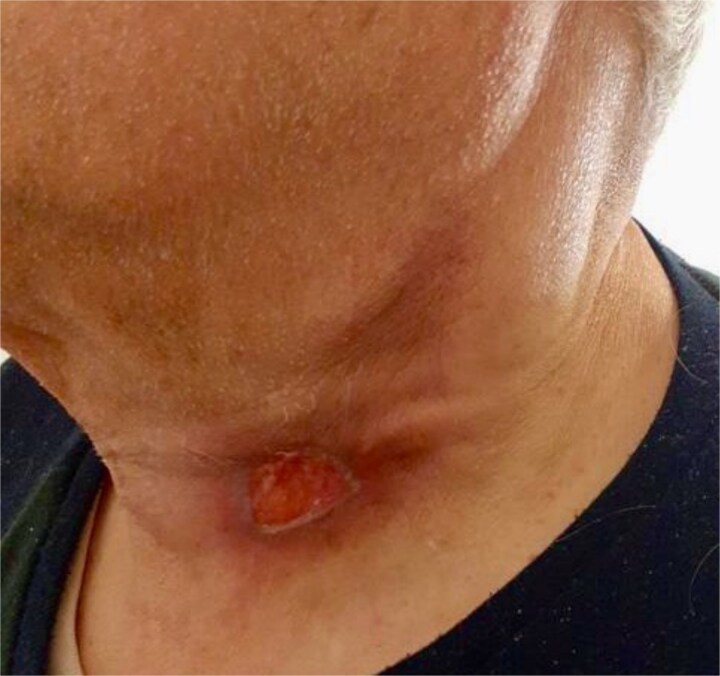
Clinical status following antituberculosis treatment.

Over the course of treatment, the patient demonstrated a progressive reduction in the size of the cervical lesion, accompanied by resolution of associated symptoms. By the end of the therapy, the lymphadenopathy had significantly regressed, leaving only residual fibrotic changes (Fig. 2). No evidence of recurrence or systemic dissemination was observed during follow-up.

This case highlights the importance of considering tuberculous lymphadenitis in the differential diagnosis of rapidly enlarging cervical masses, particularly in elderly patients with prior oncological histories where the clinical suspicion may strongly favor malignancy. Timely recognition, accurate diagnosis through aspiration biopsy, and adherence to guideline-based therapy are crucial to achieving favorable outcomes, even in complex patients with multiple comorbidities.

## Discussion

Cervical lymphadenitis is a common clinical entity that can develop secondary to a wide range of causes, including traumatic injury, infections, inflammatory processes, congenital masses, and both benign and malignant pathologies. Among infectious causes, *M. tuberculosis* remains the most important, given its prevalence and potential for systemic complications. TB continues to represent a major global health threat. According to World Health Organization (WHO) data, in 2018, ~10 million people were diagnosed with TB globally, and 1.5 million deaths were attributed to the disease. Of these cases, 15%–20% were EPTB, with cervical lymph nodes being the most frequently involved site, reported in 60%–90% of tuberculous lymphadenopathy cases [3].

Clinically, TB most often presents with systemic symptoms such as fever, night sweats, weight loss, and diaphoresis [7]. The differential diagnosis of cervical lymphadenopathy is broad and includes other infectious conditions such as nontuberculous mycobacterial lymphadenitis, toxoplasmosis, cat-scratch disease, and fungal infections, as well as noninfectious causes such as sarcoidosis and malignant neoplasms. This wide spectrum emphasizes the diagnostic challenge of scrofula, particularly in elderly patients or those with previous oncological history, where suspicion of malignancy is high [8].

FNAC is widely used as a minimally invasive initial investigation, with reported sensitivities between 70% and 85% for diagnosing tuberculous lymphadenitis. However, as seen in our case, FNAC may occasionally lead to inconclusive results, requiring repeat aspiration or excisional biopsy. The presence of caseous necrosis and purulent aspirate remains pathognomonic for TB and was critical in establishing the definitive diagnosis [9].

Treatment is primarily medical, consisting of a standard multidrug ATT regimen, with surgery reserved for complications such as abscesses, sinus formation, or diagnostic uncertainty [6]. Our patient responded favorably to the 6-month ATT course, with significant regression of the cervical lesion and no recurrence on follow-up, despite advanced age and comorbidities.

## Conclusion

CTL (scrofula) remains a diagnostic challenge due to its ability to mimic malignant and other infectious conditions. This case highlights the importance of a differential diagnosis when evaluating cervical lymphadenopathy, particularly in elderly patients with a history of malignancy where the likelihood of cancer is high. Fine-needle aspiration and biopsy with demonstration of caseous necrosis were fundamental in establishing the correct diagnosis. Early initiation of guideline-based ATT led to favorable outcomes despite advanced age and multiple comorbidities. Clinicians should remain vigilant for scrofula as a potential etiology of neck masses, ensuring timely diagnosis and treatment to prevent complications and unnecessary interventions.
